# Comparison of Regional Brain Perfusion Levels in Chronically Smoking and Non-Smoking Adults

**DOI:** 10.3390/ijerph120708198

**Published:** 2015-07-16

**Authors:** Timothy C. Durazzo, Dieter J. Meyerhoff, Donna E. Murray

**Affiliations:** 1 Department of Radiology and Biomedical Imaging, University of California, San Francisco, CA 94143, USA; E-Mails: dieter.meyerhoff@ucsf.edu (D.J.M.); donna.murray@ucsf.edu (D.E.M.); 2 Center for Imaging of Neurodegenerative Diseases, San Francisco VA Medical Center, CA 94121, USA

**Keywords:** cigarette smoking, nicotine, brain perfusion, arterial spin labeling, brain blood flow, magnetic resonance

## Abstract

Chronic cigarette smoking is associated with numerous abnormalities in brain neurobiology, but few studies specifically investigated the chronic effects of smoking (compared to the acute effects of smoking, nicotine administration, or nicotine withdrawal) on cerebral perfusion (*i.e.*, blood flow). Predominately middle-aged male (47 ± 11 years of age) smokers (*n* = 34) and non-smokers (*n* = 27) were compared on regional cortical perfusion measured by continuous arterial spin labeling magnetic resonance studies at 4 Tesla. Smokers showed significantly lower perfusion than non-smokers in the bilateral medial and lateral orbitofrontal cortices, bilateral inferior parietal lobules, bilateral superior temporal gyri, left posterior cingulate, right isthmus of cingulate, and right supramarginal gyrus. Greater lifetime duration of smoking (adjusted for age) was related to lower perfusion in multiple brain regions. The results indicated smokers showed significant perfusion deficits in anterior cortical regions implicated in the development, progression, and maintenance of all addictive disorders. Smokers concurrently demonstrated reduced blood flow in posterior brain regions that show morphological and metabolic aberrations as well as elevated beta amyloid deposition demonstrated by those with early stage Alzheimer disease. The findings provide additional novel evidence of the adverse effects of cigarette smoking on the human brain.

## 1. Introduction

Chronic cigarette smoking in young to older adults, ostensibly free of psychiatric comorbidies and clinically significant smoking-related diseases, is associated with multiple neurobiological deficits [1,2,3] that include abnormalities in regional brain morphology [4,5,6,7,8,9,10,11], metabolite concentrations [12,13,14,15], white matter microstructural integrity [16], and functional connectivity [17,18]. These neurobiological abnormalities appear to be most prominent in the anterior cingulate cortex (ACC), dorsal prefrontal cortex (DPFC), orbitofrontal cortex (OFC), and insula, which are cortical components of the brain reward-executive oversight system (BREOS; [19,20]); the cortical and subcortical components of the BREOS are implicated in the development, progression, and maintenance of all addictive disorders [21,22]. Neurobiological abnormalities associated with chronic smoking are also apparent in posterior regions (e.g., posterior cingulate, inferior parietal lobule, precuneus) that show morphological and metabolic aberrations, and elevated beta amyloid deposition in those with mild cognitive impairment (MCI) and early stage Alzheimer’s disease (AD) [23] (These regions hereafter are referred to as AD regions).

In addition to the neurobiological abnormalities noted above, chronic cigarette smoking is related to disturbances in brain perfusion (*i.e.*, blood flow). Cigarette combustion products contain nicotine and multiple other compounds that acutely and chronically influence cerebral hemodynamics via their indirect and direct adverse effects on cerebral vasoreactivity, vascular structural integrity, as well as through alterations in cerebral cellular metabolism [1,2,3,23]. Few studies have specifically investigated the long-term chronic effects of smoking (as opposed to the acute effects of smoking, nicotine administration, or nicotine withdrawal) on cerebral perfusion in participants without a history of clinically significant psychiatric or biomedical conditions. Early investigations reported globally decreased cortical perfusion via CT ^133^Xe inhalation in elderly smokers [24,25,26], as well as by SPECT in middle-aged smoking adults [27]. A recent fluorodeoxyglucose (FDG) PET study found that elders with a history of chronic smoking demonstrated significantly decreased cerebral glucose metabolism over a large volume of the bilateral cerebral cortex [28]; FDG uptake, an established measure of glucose metabolism, is tightly coupled with cerebral blood flow [29,30]. A limitation of these previous studies is that blood flow or glucose metabolism measurements were obtained over the entire cortical gray matter or involved an average of multiple bilateral cortical regions. Consequently, it is unclear if there are specific regional differences between chronic smokers and non-smokers in cortical perfusion. This study investigated the effects of chronic smoking on regional cerebral perfusion in young to middle-aged adults (e.g., 25–60), who represent the greatest proportion of active smokers in the United States [31]. In this study, regional cortical brain perfusion levels in predominately male, middle-aged smokers and non-smokers were compared via 4 Tesla magnetic resonance continuous arterial spin labeling.

We hypothesized that:

(1) Smokers demonstrate significantly lower perfusion than non-smokers in components that comprise the bilateral BREOS and AD regions.

(2) In smokers, greater smoking severity (*i.e.*, higher lifetime years of smoking and pack-years) is associated with lower regional cortical perfusion in components that comprise the bilateral BREOS and AD regions.

## 2. Materials and Methods

### 2.1. Participants

Healthy, community-dwelling smokers (*n* = 34; four females) and non-smokers (*n* = 27; four females) were recruited via posters, electronic billboards, and word-of-mouth. Participants were between the ages of 24 and 69 and gainfully employed at the time of study (see Table 1). Prior to study participation, all participants provided written informed consent according to the Declaration of Helsinki, and the consent document and procedures were approved by the University of California San Francisco and the San Francisco VA Medical Center.

**Table 1:** Demographic and clinical measures.

Variable	Non-Smokers (*N* = 27)	Smokers (*N* = 34)
Age (years)	47.3 ± 11.9	47.3 ± 10.5
Education (years)	16.5 ± 2.1	14.9 ± 2.1 *
Male (%)	85	87
Caucasian (%)	63	71
Body mass index	24.8 ± 3.0	27.1 ± 4.5 *
Beck Depression Inventory	3 ± 3	5 ± 4
STAI-trait	30 ± 10	34 ± 9
1-yr average drinks/month	15 ± 14	23 ± 20
Lifetime average drinks/month	18 ± 12	25 ± 14 *
FTND	NA	5 ± 2
Cigarettes/day	NA	18 ± 7
Total lifetime years of smoking	NA	30 ± 12
Pack years	NA	25 ± 14
Interval from last cigarette to scan (min)	NA	29 ± 18

Note. Mean ± standard deviation; * *p* < 0.05; FTND: Fagerström Test for Nicotine Dependence; STAI: State-Trait Anxiety Inventory, trait-score. One standard alcoholic drink contained 13.6 grams of pure ethanol.

Primary inclusion/exclusion criteria are fully presented elsewhere [32]. In summary, participants were screened and excluded for history of any neurologic (e.g., seizure disorder, neurodegenerative disorder, traumatic brain injury with loss of consciousness >5 min), general medical (e.g., endrocrine diseases, chronic obstructive pulmonary disease), vascular risk factors (Type 1 and 2 diabetes, hypertension, any cardiac function abnormalities, myocardial infarction, cerebrovascular accident, migraine headaches), and psychiatric (*i.e.*, mood, thought, anxiety, substance/alcohol use disorders) conditions/disorders known or suspected to influence brain neurobiology. All females were pre-menopausal, by self-report. Twenty-three of the non-smokers never smoked; four smoked less than 40 cigarettes during their lifetime, but they reported no cigarette/tobacco use in the 10 years prior to study. All smoking participants were actively smoking at the time of assessment, smoked at least 10 cigarettes/day for ≥5 years, with no periods of smoking cessation greater than 1 month in the 5 years prior to study. At the time of study, no smoker was involved in any pharmacological/behavioral smoking cessation program or used any other form of tobacco or electronic cigarettes. All smokers were allowed to smoke ad libitum prior to the magnetic resonance study. At the end of the study, smokers were offered smoking cessation resource literature.

### 2.2. Psychiatric, Medical, and Substance/Alcohol Consumption Assessment

Participants were administered the screening section of the Structured Clinical Interview for DSM-IV Axis I disorders, Patient Edition, Version 2.0, as well as an in-house questionnaire designed to screen for medical, psychiatric, neurological, and developmental conditions known or suspected to influence neurocognition or brain neurobiology. Participants also completed semi-structured interviews for lifetime alcohol consumption (Lifetime Drinking History, LDH) and substance use (in-house questionnaire assessing substance type, and quantity and frequency of use). From the LDH, the average number of drinks/month (one drink defined as containing 13.6 grams of pure ethanol) over 1 year prior to enrollment and average number of drinks/month over lifetime were calculated. Participants also completed self-report measures of depressive (Beck Depression Inventory, BDI) and anxiety (State-Trait Anxiety Inventory, trait form Y-2, STAI) symptomatologies, and family history of problem drinking. Smokers completed a measure of nicotine dependence level [Fagerström Test for Nicotine Dependence (FTND)] and provided information on the total number of cigarettes currently smoked per day, as well as the total number of years of smoking over lifetime. From this information, pack-years [*i.e.*, (typical number of cigarettes per day/20) x total number of years of smoking] were calculated for smokers. Prior to assessment, participants’ urine was tested for common illicit substances (e.g., tetrahydrocannabinol, opiates, cocaine, and amphetamines), and they were assessed for recent ethanol consumption via breathalyzer. No participant was positive for the above illicit substances or ethanol consumption at the time of assessment. See [33] for corresponding references for the above measures.

### 2.3. Magnetic Resonance Data Acquisition and Processing

Magnetic resonance (MR) studies were performed on a 4T Bruker MedSpec system with a Siemens Trio console (Siemens, Erlangen, Germany) and an 8-channel transmit-receive head coil; all scans were performed between 11 AM and 6 PM. Structural images were acquired with a 3D sagittal T1-weighted magnetization prepared rapid gradient echo acquisition sequence (1.0 × 1.0 × 1.0 mm^3^) and 2D axial T2-weighted turbo-spin echo sequence (0.9 × 0.9 × 3.0 mm^3^). Perfusion-weighted images were obtained via a continuous arterial spin labeling (ASL) single-shot echo-planar imaging sequence [34] with sixteen oblique-axial 5-mm-thick slices oriented parallel to the orbitomeatal line (in-plane resolution = 5.0 × 3.8 mm^2^; 1.45 mm slice gap; TR/TE = 5200/9 ms repetition/echo time; 1590 ms post-labeling delay; 90° flip angle; acquisition time approximately 7 min). Prior to initiation of the ASL perfusion sequence, all participants were instructed to remain awake with eyes closed. Structural MRI data were aligned with perfusion data using a fluid-flow warping based distortion correction algorithm that corrected images for partial volume effects. See [35] for full description of ASL processing methods. Regional cerebral blood flow images were then corrected for partial volume effects and co-aligned with FreeSurfer v5.1 segmented and parcellated volumes [36,37] to yield subject-specific perfusion averages (given in institutional units) for each FreeSurfer cortical and subcortical anatomical label. The ASL perfusion-weighted sequence was optimized for cortical gray matter, so group comparisons on perfusion of the lobar white matter and subcortical nuclei/structures are not provided in this report. The inferior 2–3 slices from all ASL datasets were removed from analyses due to generally poorer data quality in these regions. Therefore, only cortical regions in an axial plane (parallel to the orbitomeatal line), above the middle temporal gyrus, that contained at least 50% gray matter, were included in analyses. See Table 2 for BREOS, AD, and Non-BREOS/AD cortical regions included in this study.

### 2.4. Statistical Analyses

Multivariate analysis of covariance (MANCOVA) was used to compare smokers and non-smokers on regional cortical perfusion levels. Separate MANOVAs were conducted for the bilateral BREOS, AD, and Non-BREOS/AD regions. Average lifetime drinks per month, body mass index (BMI), and education were included as covariates because groups showed significant differences on these variables (see Table 1). Groups were not different on age, but given the large age range within the groups, and the association of age with perfusion in our previous studies [38], it was included as a covariate. MANCOVA omnibus and univariate main effects were considered statistically significant at *p* < 0.05. Significant main effects for smoking status (*i.e.*, smoker *vs.* non-smoker) were followed-up with t-tests (two-tailed; included the same covariates as in the univariate models) comparing smokers and non-smokers. Despite our *a priori* predictions, we adopted the conservative approach of adjusting alpha levels for follow-up t-tests corresponding to each MANCOVA for multiplicity of t-tests. A modified Bonferroni approach was employed [39] that adjusted alpha levels for follow-up t-tests on the basis of the average intercorrelation of perfusion values across regions (in the combined group of smokers and non-smokers) included in the MANOVA and the number of t-tests performed. The average intercorrelation for BREOS regions across smokers and non-smokers was *r* = 0.76; based on this intercorrelation, and 16 t-tests, the adjusted alpha level for BREOS follow-up t-tests comparing smokers and non-smokers was *p* ≤ 0.024. The average intercorrelation for AD regions was *r* = 0.84; based on this intercorrelation, and 16 t-tests, the adjusted alpha level for AD regions follow-up t-tests comparing smokers and non-smokers was *p* ≤ 0.036. The average intercorrelation for Non-BREOS/AD regions was *r* = 0.74; based on this intercorrelation, and 16 t-tests, the adjusted alpha level for non-BREOS/AD follow-up t-tests comparing smokers and non-smokers was *p* ≤ 0.022. Effect sizes (ES) for mean differences between smokers and non-smokers on regional perfusion levels were calculated with Cohen’s *d*. Associations between measures of smoking severity (*i.e.*, lifetime years of smoking and pack-years) and regional perfusion levels were evaluated with partial correlations, adjusting for age. Correlations between smoking severity measures and BREOS and AD perfusion were considered statistically significant at *p* < 0.05. Only associations of at least moderate magnitude (*i.e.*, *r* ≥ |0.30|) were reported.

**Table 2:** Cortical regions of interest.

Region	Subregion (Bilateral)
BREOS	Caudal anterior cingulate cortex
	Rostral anterior cingulate cortex
	Insula
	Caudal middle frontal gyrus
	Rostral middle frontal gyrus
	Lateral orbitofrontal cortex
	Medial orbitofrontal cortex
	Superior frontal gyrus
AD	Isthmus of cingulate
	Posterior cingulate
	Inferior parietal lobule
	Superior parietal lobule
	Precuneus
	Supramarginal gyrus
Non-BREOS/AD	Cuneus
	Frontal pole
	Paracentral
	Pars opercularis
	Pars orbitalis
	Pars triangularis
	Post central gyrus
	Precentral gyrus
	Superior temporal gyrus

BREOS: Brain reward/executive oversight system; AD: Alzheimer’s disease.

## 3. Results

### 3.1. Participant Characteristics

No significant differences were observed between smokers and non-smokers on age, BDI, STAI-trait, one-year average drinks/month, and positive family history of problem drinking (all *p* > 0.10). Groups were equivalent on frequency of Caucasians and females. Smokers had fewer years of education, greater BMI, and more lifetime average drinks/month (all *p* < 0.05). See Table 1.

### 3.2. Comparisons of Smokers and Non-Smokers on Regional Cortical Perfusion

#### 3.2.1. BREOS

**Figure 1:**
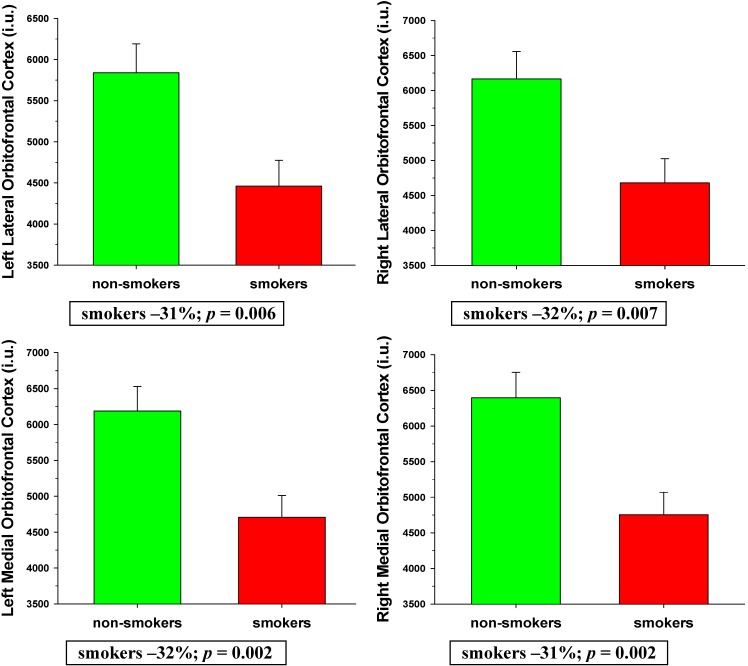
Comparisons of smokers and non-smokers on BREOS perfusion levels.

MANCOVA indicated smoking status was a significant omnibus predictor of BREOS perfusion levels [*F* (16, 42) = 2.75, *p* = 0.004]. Age, education, lifetime average drinks/month, and BMI were not significant omnibus predictors of BREOS perfusion (all *p* > 0.60). Main effects for smoking status were observed for the bilateral medial and lateral OFC (all *p* < 0.05), with trends for the right caudal middle frontal gyrus (*p* = 0.079) and right insula (*p* = 0.089). Follow-up t-tests (adjusted threshold for significance *p* ≤ 0.024) indicated smokers had significantly lower perfusion than non-smokers in the left (*p* = 0.006; *ES* = 0.76) and right (*p* = 0.007; *ES* = 0.73) lateral OFC, and the left (*p* = 0.002; *ES* = 0.84) and right (*p* = .001; *ES* = 0.89) medial OFC (see Figure 1). There were no significant interactions among smoking status and age, education, lifetime average drinks/month, and BMI. In all regions contributing to the BREOS, smokers demonstrated numerically lower perfusion than non-smokers. Findings were essentially unchanged after excluding females from the above analyses.

#### 3.2.2. AD Regions

**Figure 2:**
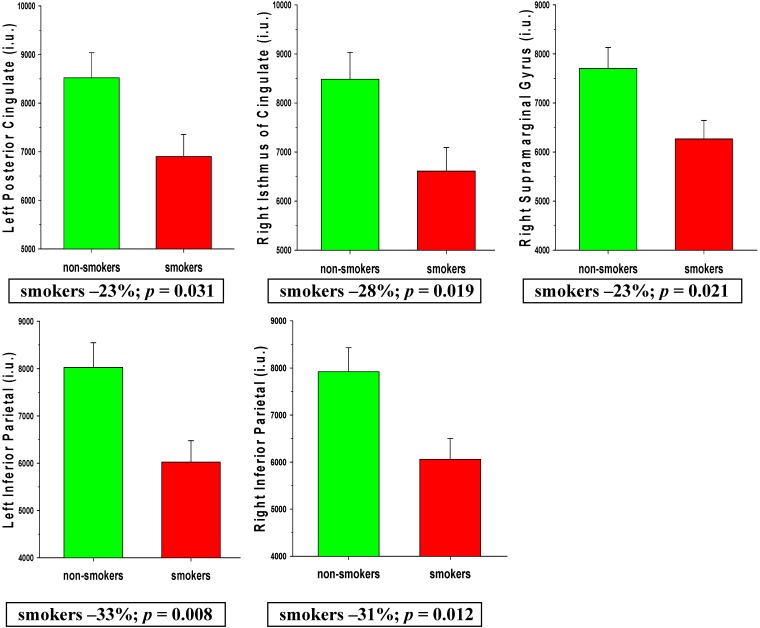
Comparisons of smokers and non-smokers on AD region perfusion levels.

MANCOVA indicated that smoking status was a significant omnibus predictor of AD regions perfusion levels [*F* (12, 46) = 2.30, *p* = 0.022]. Age, education, lifetime average drinks/month, and BMI were not significant omnibus predictors of AD regions perfusion (all *p* > 0.18). Main effects for smoking status were observed for the bilateral inferior parietal lobule, bilateral posterior cingulate, right isthmus of the cingulated gyrus, and right supramarginal gyrus (all *p* < 0.05), with trends for the left isthmus of the cingulate (*p* = 0.051), left precuneus (*p* = 0.090), and right insula (*p* = 0.084). Follow-up t-tests (adjusted threshold for significance *p* ≤ 0.036) indicated smokers showed significantly lower perfusion than non-smokers in the left posterior cingulate (*p* = 0.031; *ES* = 0.61), right isthmus of the cingulate (*p* = 0.019; *ES* = 0.66), and right supramarginal gyrus (*p* = 0.021; *ES* = 0.65), and left (*p* = 0.008; *ES* = 0.75) and right (*p* = 0.012; *ES* = 0.71) inferior parietal lobule (see Figure 2). No significant interactions were observed among smoking status and age, education, lifetime average drinks/month, and BMI. In all individual AD regions, smokers showed numerically lower perfusion than non-smokers. Findings were essentially unchanged after excluding females from the above analyses.

#### 3.2.3. Non-BREOS/AD Regions

MANCOVA indicated smoking status [*F* (18, 40) = 2.49, *p* = 0.009], and age [*F* (18, 40) = 2.57, *p* = 0.007] were significant omnibus predictors of Non-BREOS/AD regions perfusion. Education, lifetime average drinks/month, and BMI were not significant omnibus predictors of Non-BREOS/AD regional perfusion (all *p* > 0.25). Main effects for smoking status were observed for the bilateral superior temporal gyrus, right pars orbitalis, and right frontal pole (all *p* < 0.05). Follow-up t-tests (adjusted threshold for significance *p* ≤ 0.022) indicated smokers showed significantly lower perfusion than non-smokers in the left (*p* = 0.008; *ES* = 0.72) and right (*p* < 0.001; *ES* = 1.02) superior temporal gyrus, and right frontal pole (*p* = 0.003; *ES* = 0.81). There were no significant interactions among smoking status and age, education, lifetime average drinks/month, and BMI. In all Non-BREOS/AD regions, smokers demonstrated numerically lower perfusion than non-smokers.

#### 3.2.4. Associations between Regional Perfusion and Smoking Severity Measures

Greater lifetime years of smoking (adjusted for age) were associated with lower perfusion in the following regions: *BREOS*: left (*r* = −0.41; *p* = 0.010) and right (*r* = −0.37; *p* = 0.016) lateral OFC, left (*r* = −0.31; *p* = 0.040) and right (*r* = −0.32; *p* = 0.040) medial OFC, and left insula (*r* = −0.33; *p* = 0.029); *Non-AD/BREOS:* left (*r* = −0.35; *p* = 0.030) and right (*r* = −0.32; *p* = 0.035) bank of superior temporal gyrus, left cuneus (*r* = −0.30; *p* = 0.045), and left frontal pole (*r* = −0.33; *p* = 0.030). No significant relationships were observed between lifetime years of smoking and AD regions. Pack-years, FTND score, and the interval from last cigarette smoked to the MR scan were not significantly associated with perfusion in any region.

**Figure 3:**
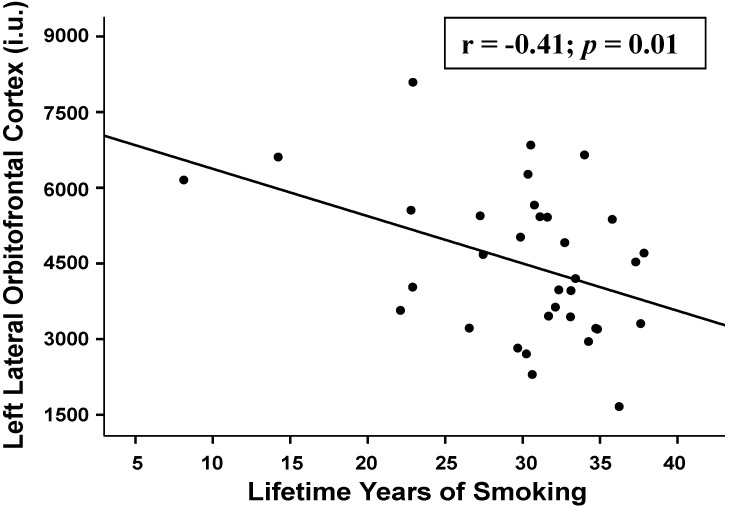
Relationship between left lateral orbitofrontal cortex perfusion and lifetime years of smoking (age-adjusted).

## 4. Discussion

The primary findings from this cohort of predominately middle-aged males were: (1) smokers demonstrated significantly lower perfusion than non-smokers in the bilateral medial and lateral OFC of the BREOS; (2) smokers showed significantly lower perfusion than non-smokers in the left posterior cingulate, right isthmus of the cingulate, right supramarginal gyrus, and bilateral inferior parietal lobule of the AD regions; (3) significantly lower perfusion in smokers in Non-BREOS/AD cortical regions was observed in the bilateral superior temporal gyrus, right pars orbitalis, and right frontal pole; (4) in smokers, greater lifetime years of smoking was significantly associated with lower perfusion in multiple BREOS and Non-BREOS/AD regions.

The overall pattern of findings suggests the perfusion deficits demonstrated by this group of smokers were largely confined to BREOS and AD regions and were not consistently lateralized to either hemisphere. Only two of the nine Non-BREOS/AD regions (bilateral superior temporal gyrus, right frontal pole) showed significantly lower perfusion in smokers, which suggests perfusion in primary sensory and motor regions was largely spared in this cohort of smokers. The differences observed between smokers and non-smokers were moderate to large in magnitude (see effect sizes in Results and percent differences in Figure 1 and Figure 2), which emphasizes the substantial degree of regional perfusion deficits demonstrated by smokers. The significantly decreased perfusion observed in smokers in the bilateral medial and lateral OFC compliments previous MR studies showing thinner OFC cortex in actively-smoking young-to-middle aged adult smokers [7], smaller OFC volume in female elders (*i.e.*, >65 years of age) [10], and significantly greater rate of OFC atrophy over two years in cognitively-normal elders with a history of smoking [40]. Similarly, the significantly lower perfusion observed in smokers in the inferior parietal lobule, posterior cingulate, isthmus of cingulate is complimentary to MR morphometric studies reporting smaller inferior parietal lobule volumes in actively-smoking middle-aged adult smokers [5], lower gray matter density in the posterior cingulate of elder smokers [9], and significantly greater atrophy rate over two years in the inferior parietal lobule, posterior cingulate, and isthmus of the cingulate in cognitively-normal elders with a history of smoking [40]. Decreased brain perfusion and atrophy in several of the above-listed regions were concurrently observed in patients with frontotemporal dementia [41], and AD [42]. These parallel findings suggest that reduced cerebral blood flow may be associated with the morphological abnormalities demonstrated by smokers.

The medial and lateral divisions of the OFC are implicated in evaluation of stimulus saliency and representation of reward magnitude, self-monitoring, regulation of emotional and affective tone, impulse control, and aspects of decision-making and executive skills [43,44,45], which are reported to be abnormal in chronic smokers [2,3,15,46,47,48,49,50]. Collectively, the inferior parietal lobule, posterior cingulate, isthmus of cingulate, and supramarginal gyrus subserve regulation of visual attention, language comprehension, memory, and processing of complex visual social and emotional cues [51,52]. The smoking-related brain morphological, metabolite and white matter microstructural abnormalities found in previous MR studies, combined with the perfusion deficits observed in smokers in the present study, may at least partially explain the dysfunction in multiple neurocognitive abilities reported in smokers across adulthood [1,28,53], as well as the greater rate of neurocognitive decline in elders with a history of smoking [1,23].

In smokers, greater lifetime years of smoking (age-adjusted) showed moderate-strength associations with decreased perfusion levels in multiple BREOS and Non-BREOS/AD regions. The pattern of findings suggests that greater smoking chronicity was related to diminished perfusion in temporal and anterior frontal lobe regions. In this study, smokers were allowed to smoke *ad libitum* prior to the MR study to mitigate against the potential effects of nicotine withdrawal on cerebral blood flow (see [53]). The plasma half-life of nicotine is approximately two hours [54], therefore, plasma nicotine levels would have accrued (e.g., two or more half-lives) with regular smoking in the hours leading up to MR studies in the late morning or afternoon [55]. Additionally, in smokers, regional perfusion levels were not significantly related to FTND score (level of nicotine dependence) or the interval from last cigarette smoked. In our previous studies of smokers with alcohol and substance use disorders, regional perfusion levels were also unrelated to the interval from last cigarette smoked to scan [38,56,57]. Therefore, it is unlikely that nicotine withdrawal in smokers significantly influenced the observed relationships between lifetime years of smoking and regional perfusion, or the regional differences observed between smokers and non-smokers.

The particulate and gas phases of cigarette smoke contain numerous compounds that may adversely affect the structural and/or functional integrity of cerebral neurons and glia, as well as that of endothelial cells forming the tunica intima and smooth muscle of the tunica media of the cerebrovasculature, and/or promote subclinical/clinically significant vascular disease that may influence cerebral hemodynamics [1,23]. We did not measure participant blood flow velocities of the cerebral distribution arteries, vasoreactivity, or capillary transit times, all of which are fundamentally related to regional brain tissue perfusion [58,59,60,61]. Consequently, it is not possible to determine if the regional cortical perfusion deficits demonstrated by the middle-aged smokers in this study are attributable to compromised neuronal and/or glial integrity, cerebrovascular dysfunction, or a combination of these factors. Additionally, while greater lifetime years of exposure to cigarettes was related to decreased perfusion in several regions, particularly in BREOS components, the magnitude of these relationships were only moderate, and no significant relationships observed between smoking severity measures and AD region perfusion. Taken together, it is possible that the perfusion deficits observed in AD regions in this sample of smokers may antedate the onset of chronic smoking and represent an endophenotype for increased risk of initiation of cigarette smoking and associated nicotine dependence.

This study has limitations that may affect the generalizability of the findings. Data quality in several mesial and lateral temporal and mesial occipital regions was not sufficient for group comparisons; therefore, it is unclear if smokers and non-smokers showed perfusion differences in other functionally important AD regions (e.g., entorhinal and parahippocampal cortex). Unrecorded premorbid/comorbid group differences in lifestyle or biomedical conditions (e.g., diet/nutrition, exercise, subclinical pulmonary, cardiovascular or cerebrovascular dysfunction) and/or genetic polymorphisms [28] may have influenced the results. Perfusion measurements were obtained with only one post-labeling delay, which may not have fully captured maximal perfusion deficits in all participants due to individual differences in arterial transit time. The sample size was modest and, given the consistent pattern of lower perfusion in smokers across all regions, a larger sample size may have resulted in increased power to detect statistically significant group differences in more regions. The small number of females precluded assessment of sex effects.

## 5. Conclusions

The predominately middle-aged smokers in this study demonstrated substantially lower cortical perfusion than non-smokers in multiple regions, most notably in two spatially distinct areas: anterior frontal regions implicated in the development, progression, and maintenance of all addictive disorders (*i.e.*, BREOS regions), as well as in posterior cingulate and parietal regions that show morphological and metabolic abnormalities, and elevated beta amyloid deposition in those with MCI and early stage AD (*i.e.*, AD regions). There is now considerable epidemiological evidence indicating that chronic smoking in adulthood is robustly associated with increased risk for AD (see [23]). AD is an insidious process, and is characterized by an extended preclinical period that may begin 20 years or more before clinically significant dementia symptomatology is exhibited [62]. It is increasingly apparent that chronic cigarette smoking in middle-aged and elder adults is associated with neurobiological abnormalities that are similar to those which emerge during the later preclinical and prodromal stages of AD (see [23]). Therefore, chronic smoking may be associated with the initiation and progression of cerebral neuropathology that places smokers at increased risk for AD. Extended smoking cessation in elders was associated with increasing global cortical perfusion in an early, small sample size, CT ^133^Xe inhalation study [26]. Thus, longitudinal investigations of the effects of smoking cessation on regional brain perfusion, with a greater number of females, are needed to assess if the pattern of regional perfusion deficits observed in smokers is persistent or improves with smoking cessation.
